# Exploring the sequence features determining amyloidosis in human antibody light chains

**DOI:** 10.1038/s41598-021-93019-9

**Published:** 2021-07-02

**Authors:** Puneet Rawat, R. Prabakaran, Sandeep Kumar, M. Michael Gromiha

**Affiliations:** 1 Protein Bioinformatics Lab, Department of Biotechnology, Bhupat and Jyoti Mehta School of Biosciences, Indian Institute of Technology Madras, Chennai, 600036 Tamil Nadu India; 2 Biotherapeutics Discovery, Boehringer-Ingelheim Inc., 5571 R & D Building, 175 Briar Ridge Road, Ridgefield, CT 06877 USA; 3 Advanced Computational Drug Discovery Unit (ACDD), Institute of Innovative Research, Tokyo Institute of Technology, 4259 Nagatsutacho, Midori-ku, Yokohama, Kanagawa 226-8501 Japan

**Keywords:** Computational biology and bioinformatics, Structural biology

## Abstract

The light chain (AL) amyloidosis is caused by the aggregation of light chain of antibodies into amyloid fibrils. There are plenty of computational resources available for the prediction of short aggregation-prone regions within proteins. However, it is still a challenging task to predict the amyloidogenic nature of the whole protein using sequence/structure information. In the case of antibody light chains, common architecture and known binding sites can provide vital information for the prediction of amyloidogenicity at physiological conditions. Here, in this work, we have compared classical sequence-based, aggregation-related features (such as hydrophobicity, presence of gatekeeper residues, disorderness, β-propensity, etc.) calculated for the CDR, FR or V_L_ regions of amyloidogenic and non-amyloidogenic antibody light chains and implemented the insights gained in a machine learning-based webserver called “V_L_AmY-Pred” (https://web.iitm.ac.in/bioinfo2/vlamy-pred/). The model shows prediction accuracy of 79.7% (sensitivity: 78.7% and specificity: 79.9%) with a ROC value of 0.88 on a dataset of 1828 variable region sequences of the antibody light chains. This model will be helpful towards improved prognosis for patients that may likely suffer from diseases caused by light chain amyloidosis, understanding origins of aggregation in antibody-based biotherapeutics, large-scale in-silico analysis of antibody sequences generated by next generation sequencing, and finally towards rational engineering of aggregation resistant antibodies.

## Introduction

Antibodies are an essential part of human immune response to invading pathogens. However, they are also involved in many diseases, such as systemic light chain amyloidosis, autoimmune disorders and plasma cell disorders (PCD), including multiple myeloma (MM), light chain deposition disease (LCDD) and Waldenstrom’s macroglobulinemia (WM)^1–4^. The studies have shown that the antibody light chains (LC) that form amyloid fibrils display inherent sequence variability and it has been difficult to predict their aggregation propensity solely from the amino acid sequence^5,6^. Researchers have used sequence-based aggregation-scoring algorithms including GAP^7^, TANGO^8^, WALTZ^9^, PASTA^10^, Aggrescan^11^, FoldAmyloid^12^, ANuPP^13^ etc. to predict the solubility and identify the aggregation hotspots within amyloid-forming proteins. These algorithms have utilized sequence and structure-based properties such as patterns of hydrophobic and polar residues, β-strand propensity, charge, ability to form cross-β motif, aggregation propensity scales determined from experimental data, solvent-exposed hydrophobic patches on molecular surface and so on. Advantages and limitations of these algorithms have been reviewed elsewhere^14^. A common wisdom emerging from these studies is that the presence of an aggregation-prone region (APR) may be a necessary but not sufficient condition for protein aggregation to occur. A number of other factors such as the location of APRs in protein structure, conformational stability of the native state, solution conditions, and kinetics of aggregation process also play major roles^15–21^. The studies performed on aggregation in antibodies have revealed that APRs can be found everywhere in their structure, including the complementarity determining regions (CDRs) as well as fragment crystallizable (Fc) regions^15,22–24^. APRs present at sequence regions overlapping with the CDRs contribute significantly towards antigen recognition^22^. Molecular dynamics studies have demonstrated that CDR overlapping APRs are more likely to initiate aggregation than the other APRs in the fragment antigen-binding (Fab) regions of antibodies^16,25^.

A major challenge with the prognosis and treatment of AL amyloidosis is high diversity of antibodies among individuals^26^. Although there are methods for high-throughput sequencing of antibody repertoires, it is not feasible to experimentally determine the amyloidogenicity for each antibody. Hence, it is necessary to develop computational algorithms for fast and accurate prediction of aggregating light chains. Computational algorithms currently available to the scientific community need improvement since they are not efficient enough to determine the solubility of the antibodies and show weak correlation with conformational stability in some cases^24^. David et al.^27^ have previously developed a method based on Bayesian classifier and decision trees to predict the light chain amyloidogenesis using sequence information. Liaw et al.^28^ proposed a method using Random Forests classifier with dipeptide composition, which discriminated amyloidogenic and non-amyloidogenic antibody light chains.

In this study, we have analyzed the amino acid sequences from variable domains (V_L_) of 348 amyloidogenic and 1480 non-amyloidogenic antibody light chains available in AL-Base^29^. These V_L_ sequences belong to both κ and λ isotypes. The sequence conservation analysis using Shannon entropy and aggregation propensity analysis using conventional aggregation related features (charge, hydrophobicity and disorderness) revealed that light chain variable (V_L_) domains of kappa (κ) isotype have lower inherent aggregation propensity but greater sequence conservation among the amyloidogenic light chains in comparison with the non-amyloidogenic ones. On the other hand, the variable domains of lambda (λ) isotype have higher inherent aggregation propensity and similar levels of sequence conservation levels within the amyloidogenic and non-amyloidogenic light chain datasets. Furthermore, we have developed a machine learning model, “V_L_AmY-Pred”, to predict amyloidogenic and non-amyloidogenic variable region (V_L_) sequences of the light chain. Our method showed a prediction accuracy of 79.7%, with an area under the curve (AUC) value of 0.88 on the complete dataset. We benchmarked other APR prediction algorithms on the antibody dataset and analyzed the aggregation propensity, APR location, and gatekeeper residues.

## Materials and methods

### Dataset used in the study

Bodi et al.^29^ have developed a database called “Amyloid light chain database” (AL-Base), which contains amino acid or translated mRNA sequences of variable region of light chain (V_L_) from patients suffering from light chain amyloidosis, multiple myeloma and other healthy Individuals. The database is classified into amyloid plasma cell disorder (AL-PCD), other plasma cell disorder (other-PCD) and non-plasma cell disorder (non-PCD) (http://albase.bumc.bu.edu/aldb). The light chain sequences classified as other-PCD and non-PCD are considered non-amyloidogenic in the current analysis. However, it is important to note that non-PCD light chains may form amyloids if their concentrations increase to levels greater than the physiological level over a period of time. Increase in concentration is unlikely to affect amyloidogenicity of other-PCD light chains, as it is already present in high concentrations in the patient’s circulatory system.

We further processed the sequences in “AL-Base” database and excluded the sequences with missing or unmatched FRs and CDRs. Isotypes of the light sequences were verified via NCBI IgBLAST^30^. The final dataset of sequences obtained from AL-Base is listed at V_L_AmY-Pred web server (https://web.iitm.ac.in/bioinfo2/vlamy-pred/) under “Dataset used in the study” section. It contains 348 (19%) amyloidogenic and 1480 (81%) non-amyloidogenic V_L_ sequences.

A test set (AL-Test) was prepared to develop a machine learning-based classification model by randomly taking 10% of the amyloidogenic and non-amyloidogenic sequences from the AL-Base dataset. In addition, the test dataset used by David et al.^27^ was used as a blind test set in our study. This blind test set contains 103 amyloidogenic and 28 non-amyloidogenic light chain sequences. Moreover, V_L_ domain sequences from 242 clinical-stage antibody therapeutics (CSTs) and 14,037 antibody sequences collected by Raybould et al.^31^ from human antibody repertoires were also used to identify potential aggregation nucleating V_L_ domains.

### Sequence conservation of variable region (V_L_) of the light chain

We carried out multiple sequence alignment (MSA) and generated consensus sequences for kappa (κ) and lambda (λ) isotypes using MAFFT^32^. The Shannon entropy and consensus sequences were calculated for the aligned sequences using Bio3D package in statistical language R^33^, and occupancy of residues at a particular position was taken from the Jalview^34^.

### Assessment of aggregation related features

The hydrophobicity, presence of gatekeeper residues (D, E, R, K and P) and disorderness features were assessed for the CDRs and FRs in V_L_ domains. The hydrophobicity scale (H_nc_, normalized consensus hydrophobicity) was taken from the literature^35^, and residue-wise protein disorderness was calculated from IUPred2A server^36^. The average values were calculated for each region of V_L_ sequences using Eq. (1).

1 $${F}_{avg}=\frac{{\sum }_{i=1}^{N}{F}_{i}}{N}$$

where ${F}_{avg}$ is the average value of the feature for the V_L_-region/FR region/CDR region, ${F}_{i}$ is feature value for the ith residue present in the respective region and N is the length of the region.

### Development of machine learning-based classification model

A machine learning model was developed to classify amyloidogenic and non-amyloidogenic antibodies. The classification model was trained on 313 amyloidogenic and 1332 non-amyloidogenic sequences of AL-Base dataset (10% sequences were set aside for the AL-Test set as described above in “Dataset used in the study”).

#### Collection of features

The features used in the development of classification model include 70 single amino acid features from AAIndex database^37^ and literature^38^ (Supplementary Table S1). These single amino acid features were averaged for the variable region (V_L_-region), complementarity determining regions (CDRs) and framework regions (FRs) using Eq. (1). The CDR and FR information for each light chain variable domain was taken from the “AL-Base” server and follows IMGT numbering scheme. The other features used in the model development include 11 features calculated from online servers related to solvent accessibility, secondary structure propensity and aggregation propensity^11,39^; 9 sequence composition features (charge, polar, non-polar and aromatic residues); and features used by PAGE (symmetric charge, aromaticity and β-sheet propensity)^40^ (Supplementary Table S2).

#### Attribute selection and classification

Several feature selection and classification methods were employed in Weka^41^ to efficiently classify the AL-Base dataset. The final model used a decision tree algorithm called “PART” for the classification of aggregating and non-aggregating light chain variable region sequences. “PART” algorithm uses the “separate-and-conquer” method, and builds a partial decision tree using “C4.5” algorithm in each iteration to choose the best decision tree. The threshold for the classifier was manually optimized to 0.15 using “ThresholdSelector” in Weka to maintain the trade-off between sensitivity and specificity, which occurred due to class imbalance. The unpruned parameter was kept “True” for the “PART” algorithm and all other parameters were kept default.

#### Performance evaluation

The performance of the classification model was measured mainly using area under the receiver operating characteristic (ROC) curve values due to class biasness (348 amyloidogenic VL domain sequences versus 1480 non-amyloidogenic ones). ROC curve is a plot between true positive rate and false positive rate and estimates the trade-off between sensitivity and specificity at different thresholds. Hence, class imbalance does not affect the area under the ROC curve values. The robustness of the model is evaluated using leave-one-out cross-validation, where n-1 data used for the training and tested on the remaining one, recursively. We estimated the following performance measures after optimizing the threshold for the final model:

2 $$Accuracy=\frac{TP+TN}{TP+TN+FP+FN}$$

3 $$Sensitivity=\frac{TP}{TP+FN}$$

4 $$Specificity=\frac{TN}{TN+FP}$$

where TP, TN, FP and FN are number of true positives, true negatives, false positives and false negatives, respectively. Here, amyloidogenic light chain dataset is considered positive class, and non-amyloidogenic light chain dataset is considered negative class.

#### Web server development

A webserver entitled “V_L_AmY-Pred” (prediction of amyloidogenic antibody light chain variable domains) has been developed for the classification of amyloidogenic and non-amyloidogenic V_L_-region sequences. The FRs and CDRs in the V_L_-region are annotated by ANARCI^42^ tool in the webserver using IMGT numbering^43^. The webserver takes the V_L_-region of the antibody as an input and predicts the amyloidogenic/non-amyloidogenic nature of the sequence. The webserver also generates aggregation profile for each input using an in-house aggregation propensity prediction server called “ANuPP”^13^. The V_L_AmY-Pred web server is freely available and can be accessed at https://web.iitm.ac.in/bioinfo2/vlamy-pred/.

#### Comparison with APR prediction algorithms

The TANGO^8^ and WALTZ^9^ aggregation-prone region (APR) prediction algorithms were used to analyze and compare the aggregation propensity values of the V_L_ domain sequences, position of aggregation-prone regions (APR) in the V_L_ sequence, aggregation propensity of the APRs, presence of gatekeeper residues (D, E, R, K and P) in ± 3 residues flanks of the APRs in amyloidogenic and non-amyloidogenic light chain dataset.

## Results and discussion

### Sequence conservation in light chain variable domains (V_L_)

**Figure 1:**
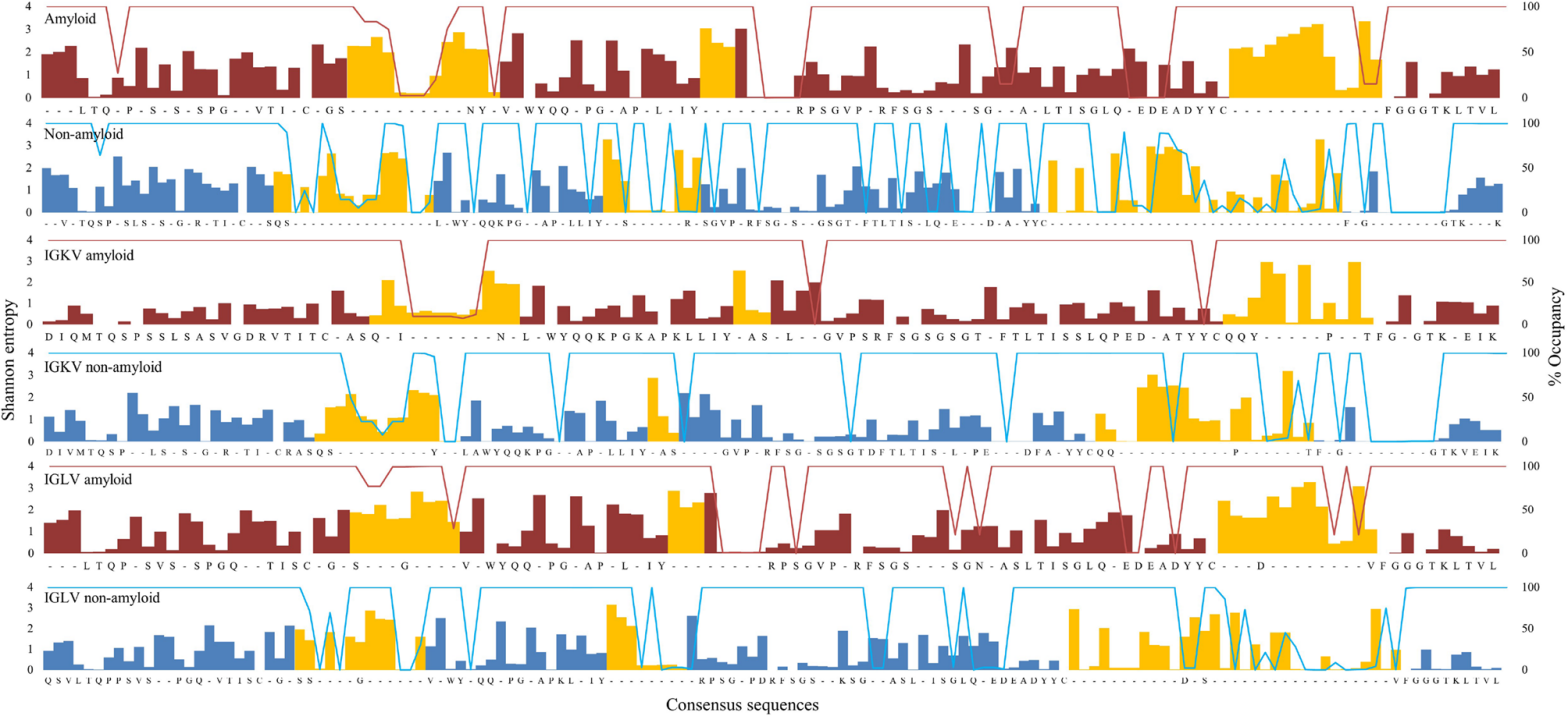
Residue wise Shannon entropy and occupancy plotted for consensus sequences from amyloidogenic (red) and non-amyloidogenic light chains (blue) of complete dataset, kappa (κ) isotype and lambda (λ) isotype. The bar graph shows the Shannon entropy (left axis) and the line graph (right axis) shows the percent occupancy. The CDR regions in the consensus sequence (x-axis) are colored in yellow. Low occupancy values denote more gaps in the multiple sequence alignment.

The dataset containing the lambda (λ) light chain sequences has a significantly greater proportion of amyloidogenic sequences compared to those of the kappa (κ) isotype (Supplementary Fig. S1). The sequence conservation of the light chains was analyzed for the whole dataset as well as for kappa (κ) and lambda (λ) isotypes using Shannon entropy (Fig. 1, Supplementary Table S3). The lower value of Shannon entropy means greater conservation and vice versa. As expected, overall FRs were relatively more conserved compared to CDRs in AL-Base dataset. The amyloidogenic light chains in the kappa (κ) dataset show higher sequence conservation, even in CDRs, when compared with the non-amyloidogenic ones. However, lambda (λ) isotype had almost similar sequence conservation levels in amyloidogenic and non-amyloidogenic light chains (Supplementary Table S3). The higher sequence conservation in amyloidogenic kappa light chain may be linked to low inherent aggregation propensity in kappa chain (as discussed in detail in “Comparison of kappa (κ) and lambda (λ) classes”).

### Analysis of conventional aggregation related features

The core aggregation-related features such as hydrophobicity, presence of gatekeeper residues and disorderness were analyzed for FRs and CDRs in the variable region of light chain for their role in amyloidogenicity.

#### Hydrophobicity of the CDR region

**Figure 2:**
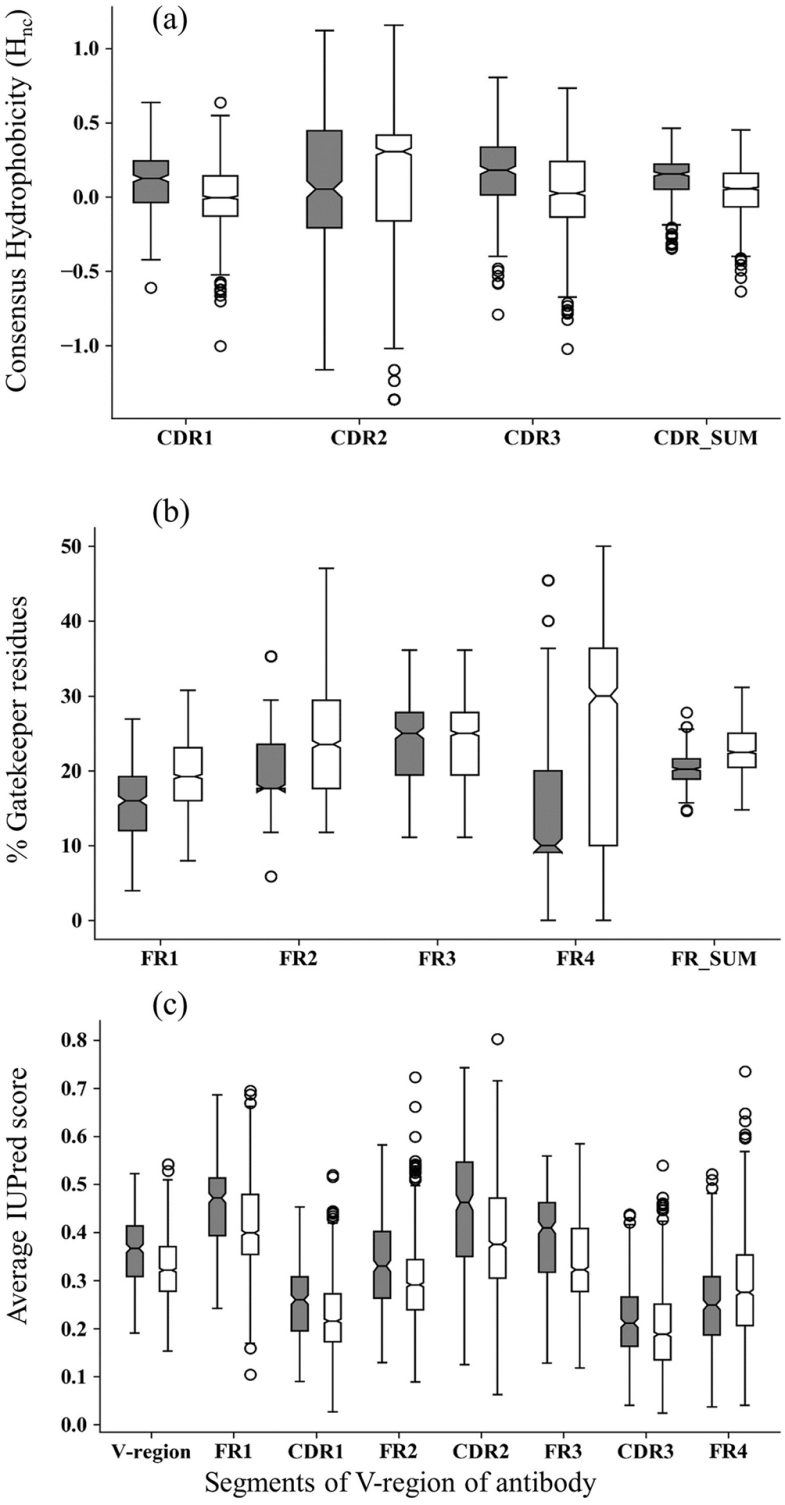
The aggregation related features (**a**) consensus hydrophobicity of the CDRs, (**b**) percentage of gatekeeper residues in FRs and (**c**) disorderness score (from IUPred2A server) calculated for different segments of V_L_-region of amyloidogenic (grey) and non-amyloidogenic (white) antibodies.

The process of aggregation in proteins generally initiates at the solvent exposed hydrophobic surface patches^44^. CDRs of the antibodies are exposed to the surface and constitute the paratope, which is involved in antigen binding. The average hydrophobicity (H_nc_)^35^ of the CDR regions in the amyloidogenic light chains was found to be greater than the hydrophobicity of the CDR regions in the non-amyloidogenic light chains (Fig. 2a). The low p-values for all the CDRs (except for CDR2) from the t-test shows difference in average hydrophobicity for amyloidogenic and non-amyloidogenic light chains was statistically significant (Supplementary Table S4). The hydrophobicity of the CDR2 had high p-value (p-value = 0.16) since most of them were just three residues long (Fig. 1).

#### Gatekeeper residues in the FR region

The presence of gatekeeper residues (D, E, R, K and P) near the aggregation-prone regions greatly hinders the aggregation capability of the proteins^45^. Several previous analysis has shown that the APR regions often overlaps with the CDR regions of the antibodies^15,22–24^. Hence, we checked the presence of the charged and beta strand breaking residues residues in the FR regions, which flanks the CDR regions (Fig. 2b). The analysis revealed that the percentage of gatekeeper residues in the FR is greater in the non-amyloidogenic antibodies than amyloidogenic antibodies and these differences are statistically significant (Supplementary Table S4). FR3 regions are the only exception, with almost a similar percentage (~ 24%) of gatekeeper residues in amyloidogenic and non-amyloidogenic light chains.

#### Disorderness of the V_L_-region

Recently, several studies have correlated protein disorderness with protein aggregation and diseases. It has been proposed that the formation of amyloid requires the destabilization of amyloidogenic globular protein for the structural rearrangement to form fibrils^46–48^. The FRs, CDRs and V_L_-region showed that amyloidogenic light chains have higher disorderness propensity compared to non-amyloidogenic light chains (FR4 being the only exception, Fig. 2c). All the predicted disorderness values for amyloidogenic and non-amyloidogenic light chain datasets were statistically significant (Supplementary Table S4).

### Comparison of kappa (κ) and lambda (λ) classes

**Figure 3:**
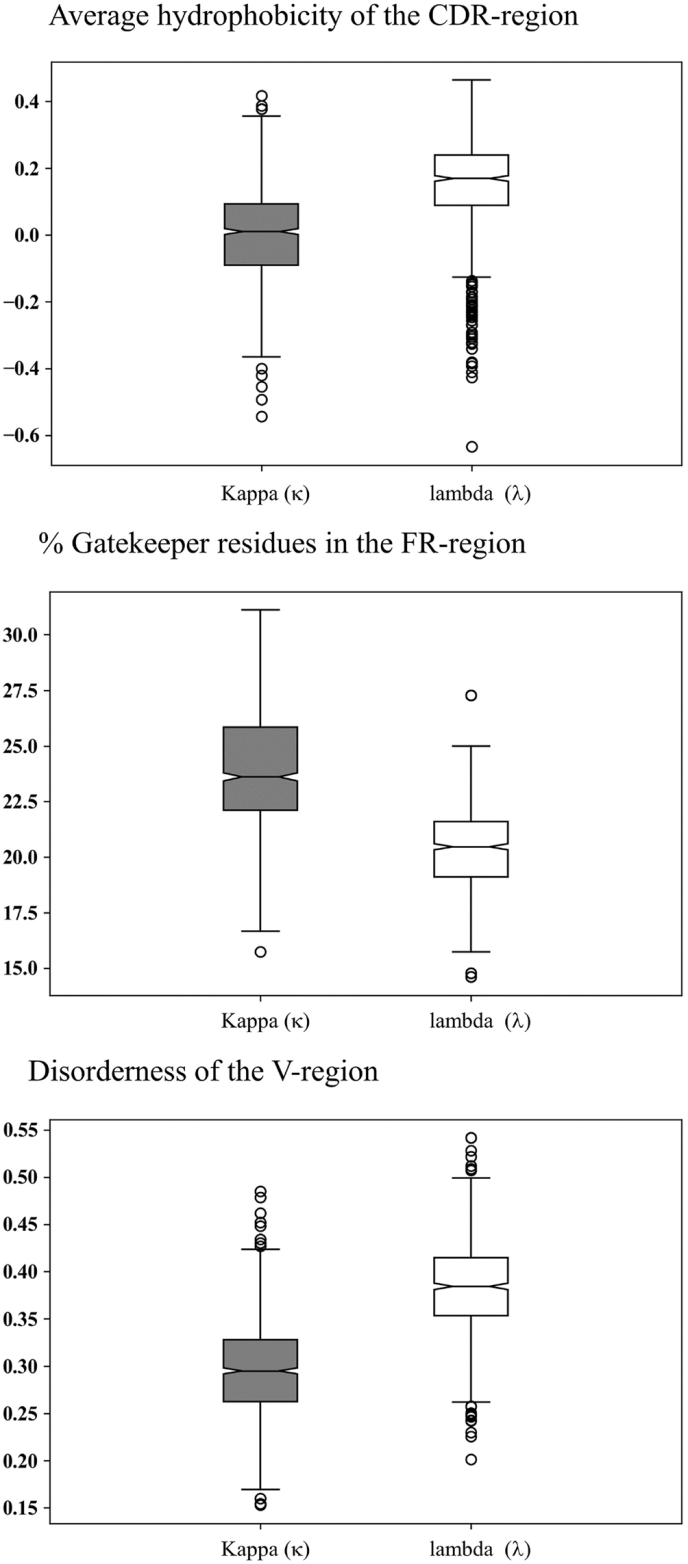
The aggregation-related features (**a**) average hydrophobicity of the CDRs, (**b**) percentage of gatekeeper residues in FRs and (**c**) disorderness score (from IUPred2A server) calculated for kappa (grey) and lambda (white) isotypes.

The features such as hydrophobicity, presence of gatekeeper residues and disorderness are classical aggregation related features which have been calculated for biologically relevant region of the light chain variable domain of antibodies. The two features, hydrophobicity of the CDRs and the gatekeeper residues in the FRs, were able to classify the light chain dataset into amyloidogenic and non-amyloidogenic with 69.6% accuracy (Supplementary Fig. S2). We analyzed the above three aggregation-related features for the kappa (κ) and lambda (λ) dataset and observed that the lambda (λ) dataset has greater aggregation capability than the kappa (κ) dataset (Fig. 3). However, as discussed in “Sequence conservation in light chain variable domains (V_L_)”, the amyloidogenic light chain dataset of kappa (κ) has higher sequence conservation, which suggests that kappa (κ) potentially requires more sequence conservation to exhibit amyloidogenicity due to low inherent aggregation capability. On the other hand, the lambda (λ) dataset has a higher inherent aggregation capability. Therefore, they might not be showing any sequence conservation (Supplementary Table S3). Our dataset also shows a similar tendency since ~ 75% of the amyloidogenic light chain sequences belong to the lambda (λ) dataset.

### Development of classification model and feature analysis

A machine learning model was developed further to classify the amyloidogenic and non-amyloidogenic V_L_-region of antibodies using the sequence-based features. The model has a prediction accuracy of 81.9% (sensitivity: 82.4% and specificity: 81.8%) with ROC value of 0.9 on the training dataset (Table 1). A set of 7 features were selected in the final model, which include three features already discussed in “Analysis of conventional aggregation related features” (hydrophobicity of the CDRs, percentage gatekeeper residues in the FRs and disorderness of the V_L_-region) and four new features, namely, β-propensity of the V_L_-region^40^, incidence of non-polar residue (A, G, I, L, M, P, V) in V_L_-region, charge transfer capability of CDRs (AAIndex Id: CHAM830107)^49^ and transfer free energy to surface for FRs (AAIndex Id: BULH740101)^50^. The highest inter-property correlation was obtained between disorderness and transfer free energy to surface (r = 0.67; Supplementary Fig. S3).

**Table 1:** Performance of the classification model for distinguishing between amyloidogenic and non-amyloidogenic variable domain light chain of antibodies.

Performance	Accuracy	Sensitivity	Specificity	ROC
Self-consistency	81.9	82.4	81.8	0.9
Leave one out CV	80	75.1	81.2	0.83
10-fold CV	72.7	78	71.4	0.82
Resampling	71.7 ± 6.9	77.2 ± 9.9	70.4 ± 9.9	0.82 ± 0.04
Test set (AL-Test)	71	65.7	72.3	0.74
Test set (David et al.)^27^	73.2	77.3	57.7	0.66
Novel germline prediction^28^	65.2 ± 11.5	62.2 ± 33.6	45.4 ± 29.4	0.65 ± 0.16
Final model	79.7	78.7	79.9	0.88
CST dataset	75.6	–	–	–
Human antibody repertoire	94.1	–	–	–

The standard deviation is mentioned for resampling and Novel germline prediction next to the performance measure. The final model was developed on complete AL-Base dataset (1828 sequences). The percent accuracy for CST dataset and human antibody repertoire represent the percentage of sequences predicted as non-amyloid.

Contrary to the common notion, it was observed that the amyloidogenic light chain dataset does not favor higher β-propensity (Supplementary Fig. S4). The plausible reason could be that antibodies are mainly composed of β-sheets and loops already, and amyloid formation requires structural rearrangement, which can be achieved by destabilizing these inherent β-sheets in the antibody. The charge transfer capability denotes the higher presence of residues (D, E, N, Q) with charge transfer accepting group (–COOH or –CONH_2_) in the CDR regions for the amyloidogenic light chain dataset. These residues also have lower probability of being present in the β-sheet^49^. The presence of the charge accepting group at the exposed CDR region might help interaction with CDRs of another antibody. The non-polar residue composition of the V_L_-region is slightly greater in the non-amyloidogenic light chain dataset (p-value: < 0.0001, Supplementary Fig. S4), which supports the above statement that polarity might help in interaction among antibody sequences to initiate aggregation. Bull et al.^50^ evaluated the transfer free energy of amino acids to surface experimentally to develop a hydrophobicity scale. Higher values of “average transfer free energy to surface” of the FR region show that these regions have a higher tendency to be exposed, which is required for the structural rearrangement during amyloid formation (Supplementary Fig. S4). It also supports the observation of beta propensity and charge transfer capability features.

### Performance of the model

The robustness of the model is evaluated using leave-one-out cross-validation (LOOCV), 10-fold cross-validation (10-fold CV), resampling and test sets (Table 1). The model has achieved significant accuracy values for LOOCV (80%) and 10-fold CV (72.7%). In resampling approach, we randomly resampled the dataset of 1828 sequences 5000 times without replacement. In each iteration, 90% of the randomly sampled data from both amyloidogenic and non-amyloidogenic light chain datasets were used in the model development, and the performance of the model was tested on the remaining 10% data. An average accuracy of 71.7% with sensitivity and specificity of 77.2% and 70.4% was obtained for the tested data, respectively. The performance of the resampling is equivalent to the performance of the test set and 10-fold cross-validation (Table 1).

**Figure 4:**
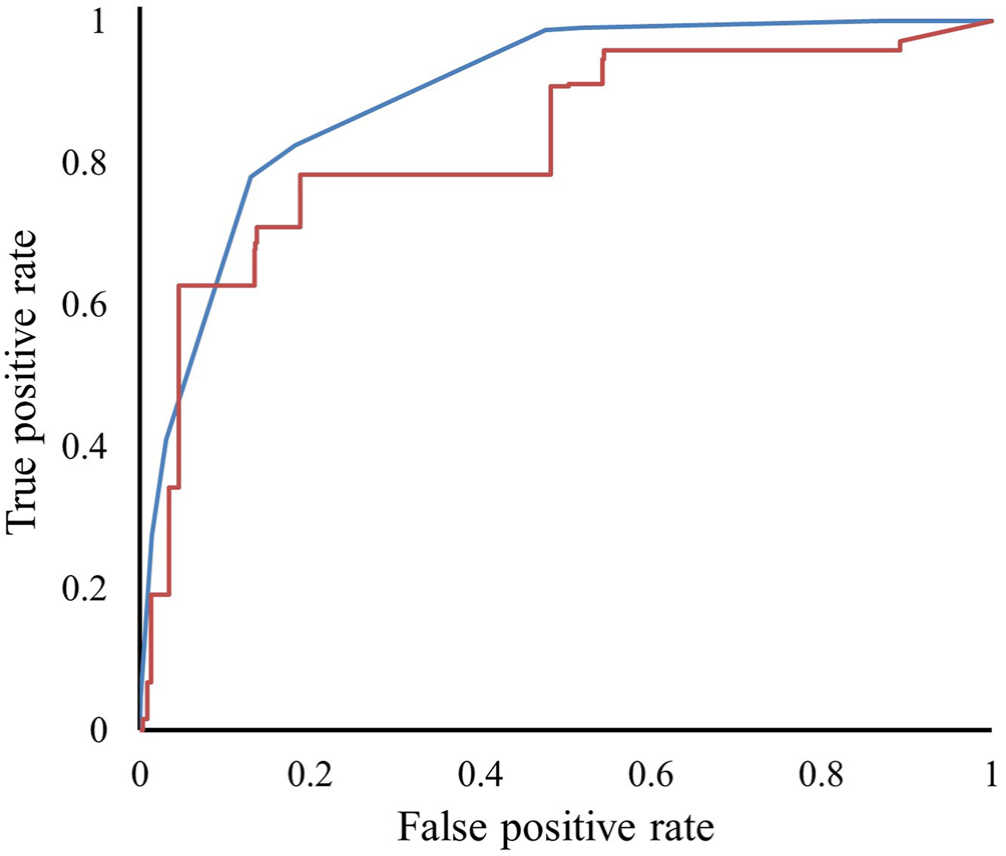
The receiver operating characteristic (ROC) curve plotted for the classification model using training dataset (blue) and leave-one-out cross-validation (red).

The ROC curves were also plotted for the training dataset (AUC: 0.9) and LOOCV (AUC: 0.83) (Fig. 4). The importance of the features is calculated by measuring the model performance after removing the respective feature or using a single feature in the model (Supplementary Table S5). Percentage of gatekeeper residues in the FR regions (ROC: 0.86) and charge transfer capability of CDRs (ROC: 0.86) are the most important features since they reduce the area under the ROC curve significantly upon removing the respective features from the model. The performance of the model was evaluated on AL-Base test dataset (AL-Test) containing 183 sequences (35 amyloidogenic and 148 non-amyloidogenic), out of which 71% or 130 (23 amyloidogenic and 107 non-amyloidogenic) sequences were predicted correctly (Table 1).

### Comparison with other methods

The two aggregation-prone region prediction algorithms, TANGO^8^ and WALTZ^9^, were used to classify amyloidogenic and non-amyloidogenic light chain variable regions of antibodies. We have analyzed the aggregation propensity, presence of aggregation-prone regions and presence of gatekeeper residues in detail for V_L_-regions (See supplementary information under the section, “Performance of aggregation-prone region prediction algorithm”). Briefly, the analysis showed that WALTZ predicted almost 3.4 times more APRs than TANGO algorithm. There was no significant difference in aggregation propensities and positions of APRs in amyloidogenic and non-amyloidogenic light chain datasets (Supplementary Fig. S5–S7, Supplementary Table S6). However, the presence of gatekeeper residues flanking the APRs showed that the amyloidogenic light chain dataset contains more APRs without gatekeeper residues in the ± 3 residue flanks (Supplementary Table S7). A significant number of APRs were also observed in the FR3 region, as reported by previous studies^51^. This region is located close to CDR regions and sometimes contributes to antigen binding^52^. FR3 region also contains a higher percentage of gatekeeper residues in both amyloidogenic and non-amyloidogenic light chain datasets, potentially to suppress these aggregation-prone regions (See Supplementary information under the section, “Analysis of FR3 region”). However, analysis of flanking residues around the APRs present in FR3 region showed that the amyloidogenic light chain dataset had more APRs without gatekeeper in the ± 3 residue flank.

The direct comparison of the TANGO and WALTZ is not appropriate with our method since APR prediction algorithms are developed for predicting the APR stretches in the protein sequences, which does not necessarily conclude that protein is amyloidogenic. A machine learning-based web server, “RFAmyloid”, is also considered in the analysis, which is developed for the prediction of amyloidogenic proteins^53^. V_L_AmY-Pred has better overall performance than the above-discussed methods and presents a better trade-off between sensitivity and specificity (Table 2).

**Table 2:** Performance of aggregation-prone region prediction algorithms (TANGO and WALTZ), RFAmyloid and V_L_AmY-Pred on AL-Base dataset (1828 sequences).

	TANGO	WALTZ	RFAmyloid	V_L_AmY-Pred
Accuracy (%)	48.4	22.2	19	80
Sensitivity (%)	34.5	96	100	75.1
Specificity (%)	51.7	4.8	0	81.2

The presence of APRs in the variable region of light chain is considered amyloidogenic for TANGO and WALTZ. The V_L_AmY-Pred results are based on Leave one out cross-validation.

The performance of V_L_AmY-Pred was also evaluated on the test dataset used by previously developed sequence-based light chain amyloidogenesis prediction models. A model developed by David et al. has shown the test set accuracy of 61.2% using Bayesian classifier on 103 amyloidogenic and 28 non-amyloidogenic sequences^27^. V_L_AmY-Pred showed the prediction accuracy of 73.2% (sensitivity: 77.3% and specificity: 57.7%) on the same dataset (97 amyloidogenic and 26 non-amyloidogenic sequences, after removing sequences not annotated as V_L_ by ANARCI software) (Table 1).

Liaw et al.^28^ validated the performance of their model “AbAmyloid” on novel germline, where they trained the model on 11 germlines and tested the performance on the remaining one germline (novel germline). AbAmyloid obtained an average performance of 72.2% on 12 germlines. We also evaluated each germline individually as a test set and obtained an average prediction performance of 65.2% (Table 1). Although, there was a high variation in the performance measures due to the inconsistent ratio of sequences (i) among all germlines and (ii) among amyloidogenic and non-amyloidogenic sequences within germline.

Monoclonal antibody candidates with a potential therapeutic application(s) are rigorously tested for solubility, non-specific protein–protein interactions, thermal unfolding and aggregation before moving to clinical trials. Hence, we have tested a set of 242 clinical-stage antibody therapeutics (CSTs) collected from literature^31^. V_L_AmY-Pred predicted 75.6% of the light chain variable region of the monoclonal antibodies as non-amyloidogenic (Table 1). The human antibodies have naturally evolved to be less amyloidogenic in physiological conditions. Hence, 14,037 light chains from the human antibody sequences obtained via NGS^31^ (75.9% kappa isotype and 24.1% lambda isotype) were also tested with our model. V_L_AmY-Pred predicted 13,208 (94.1%) light chain sequences as non-amyloidogenic (Table 1).

### Potential applications

Amyloid light chain (AL) amyloidosis affects a wide range of organs, including kidney, peripheral nervous system, heart, lungs, skin, etc., and leads to the destruction of tissues^54^. However, the mechanism of amyloid formation and underlying properties are not very well understood. There are chemotherapy and stem cell therapies available to prolong the survival of AL amyloidosis patients. However, these therapies, to a great extent, depend on the early detection of AL amyloidosis. The diagnosis methods currently available are blood and urine test using amyloid-specific dyes such as Congo Red and Thioflavin-T. We have developed V_L_AmY-Pred, an antibody-specific amyloidogenicity prediction algorithm that has a potential in-silico application as a prognosis tool for AL amyloidosis.

This machine learning model can also be used in in-silico screening of the potential amyloidogenic light chains to assist the development of therapeutic monoclonal antibodies. Monoclonal antibodies are excellent therapeutics for treating cancers, autoimmune diseases and other metabolic disorders due to their high binding specificity and affinity^55^. However, their aggregation during purification and delivery has been a major hurdle in their development.

## Conclusion

Antibodies forming aggregates are involved in many diseases and they are also a major challenge in the development of therapeutic antibodies. Multiple studies have tried to decipher the mechanism, relevant properties causing aggregation. Here, we have analyzed the sequence features of the amyloidogenic and non-amyloidogenic light chain variable regions of antibodies. The lambda (λ) isotype inherently showed higher aggregation propensity in terms of classical aggregation-related features. The key observation in the aggregation capability analysis due to common architecture of antibodies includes (i) the hydrophobicity of the CDR region (probable exposed aggregation-prone regions) in amyloidogenic light chains is higher, (ii) the percentage of gatekeeper residues is higher in FR region (flanks of the CDR regions) of non-amyloidogenic light chains. (iii) The disorderness in variable region (V_L_) is higher for amyloidogenic light chains. The sequence conservation analysis showed that the amyloidogenic light chain dataset in kappa (κ) had relatively higher sequence conservation, potentially, to maintain the amyloidogenicity. TANGO and WALTZ prediction results on the antibody dataset were very ambiguous. However, they showed that most of the APRs were present in CDR1-FR2, FR2-CDR2 and FR3 regions. A higher percentage of gatekeeper residues evolutionally suppressed the elevated presence of the APRs in the FR3 region. However, almost half of the predicted APRs in the amyloidogenic light chain dataset were not flanked by any gatekeeper residues in the FR3 region. The insights gained from the analysis were further used in the development of a machine learning model, “V_L_AmY-Pred” that can classify the amyloidogenic and non-amyloidogenic light chain sequences.

## Supplementary Information

Supplementary Information.

## Abbreviations

APR: Aggregation-prone regions
ROC: Receiver operating characteristic
FR: Framework regions
CDR: Complementarity determining regions
V_L_: Variable region of light chain
LOOCV: Leave-one-out cross-validation
