# Supplementary material for: Exploring the sequence features determining amyloidosis in human antibody light chains
Source: Sci Rep. 2021 Jul 2;11:13785. doi: 10.1038/s41598-021-93019-9 (PMC8253744; doi:10.1038/s41598-021-93019-9)
Supplement: Supplementary file 1 — Supplementary Information. [file 41598_2021_93019_MOESM1_ESM.pdf]

Exploring the sequence features determining amyloidosis in human antibody light chains

Supplementary Information

Puneet Rawat[1], R. Prabakaran[1], Sandeep Kumar[2], M. Michael Gromiha[1,3,*]

[1] Protein Bioinformatics Lab, Department of Biotechnology, Bhupat and Jyoti Mehta School of Biosciences, Indian Institute of Technology Madras, Chennai – 600036, Tamil Nadu, India.

[2] Biotherapeutics Discovery, Boehringer-Ingelheim Inc. 5571 R & D Building, 175 Briar Ridge Road, Ridgefield, CT 06877 USA

[3] Advanced Computational Drug Discovery Unit (ACDD), Institute of Innovative Research, Tokyo Institute of Technology, 4259 Nagatsutacho, Midori-ku, Yokohama, Kanagawa 226-8501, Japan

***corresponding author**

E-mail: gromiha@iitm.ac.in

Change in proportion test:

The AL-Base dataset contains 1043 (57%) light chain sequences of kappa (κ) isotype and rest 785 (43%) sequences of lambda isotype (λ). However, lambda (λ) isotype (254 out of 348, 73%) had significantly more amyloidogenic sequences compared to kappa (κ) isotype (94 out of 348, 27%) (**Supplementary Figure S1)**. We analyzed the difference between proportions of amyloidogenic antibodies of kappa (P_1) and lambda (P_2) isotypes considering the following hypothesis:

H_0 (null hypothesis): $P_1 = P_2$

H_a (alternate hypothesis): $P_1 \neq P_2$

The significantly low p-value (2.6×10^{-36}) of two-tailed z-test rejects the null hypothesis of equal proportion of amyloidogenic V_L sequences in kappa and lambda class. Hence, the light chains of lambda isotype are relatively more susceptible to amyloid formation than the kappa isotype.

Supplementary Figure S1. The distribution of amyloidogenic and Non-amyloidogenic light chain sequences from kappa (κ) and lambda (λ) isotypes.

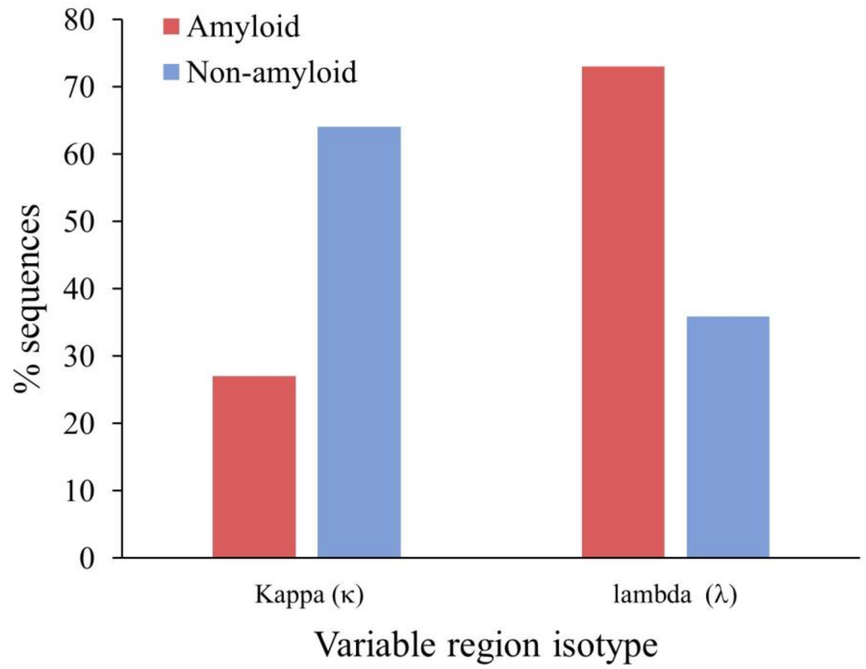

Supplementary Table S1. List of all single amino acid features. First 49 features are taken from the literature [Gromiha et al., 1999] and the rest of alphanumeric values are AAIndex ids [Kawashima et al., 2000].

Serial No.	Features*	Explanation
1	K^0	Compressibility
2	H_t	Thermodynamic transfer hydrophobicity
3	H_p	Surrounding hydrophobicity
4	P	Polarity
5	pH_i	Isoelectric point
6	pK'	The equilibrium constant with reference to the ionization property of COOH group
7	M_w	Molecular weight
8	B_l	Bulkiness
9	R_f	Chromatographic index
10	M	Refractive index
11	H_{nc}	Normalized consensus hydrophobicity
12	E_{sm}	Short and medium-range non-bonded energy
13	E_l	Long-range non-bonded energy
14	E_t	Total non-bonded energy
15	P_α	α-helical tendencies
16	P_β	β-structure tendencies
17	P_t	Turn tendencies
18	P_c	Coil tendencies
19	C_a	Helical contact area
20	F	Mean rms fluctuational displacement
21	B_r	Buriedness
22	R_a	Solvent accessible reduction ratio
23	N_s	The average number of surrounding residues
24	α_n	Power to be at the N-terminal of α-helix
25	α_c	Power to be at the C-terminal of α-helix
26	α_m	Power to be at the middle of α-helix
27	V^0	Partial-specific volume
28	N_m	Average medium contacts
29	N_l	Long-range contacts
30	H_{gm}	Combined surrounding hydrophobicity (globular and membrane)
31	ASA_D	Solvent accessible surface area for denatured
32	ASA_N	Solvent accessible surface area for native
33	ΔASA	Solvent accessible surface area for unfolding
34	ΔG_h	Gibbs free energy change of hydration for unfolding protein
35	G_{hD}	Gibbs free energy change of hydration for denatured protein

36	G_{hN}	Gibbs free energy change of hydration for native protein
37	ΔH_h	Unfolding enthalpy change of hydration
38	$-T\Delta S_h$	Unfolding entropy change of hydration
39	ΔC_{ph}	Unfolding hydration heat capacity change
40	ΔG_c	Unfolding Gibbs free energy of the chain
41	ΔH_c	Unfolding enthalpy of chain
42	$-T\Delta S_c$	Unfolding entropy changes of chain
43	ΔG	Unfolding Gibbs free energy change
44	ΔH	Unfolding enthalpy change
45	$-T\Delta S$	Unfolding entropy change
46	υ	The volume number of non-hydrogen side-chain atoms
47	s	Shape position of a branch point in a side-chain
48	f	Flexibility number of side-chain dihedral angles
49	Pf-s	Backbone dihedral probability
50	BHAR880101	Average flexibility indices
51	BIGC670101	Residue volume
52	BULH740101	Transfer free energy to the surface
53	BURA740102	Normalized frequency of the extended structure
54	CHAM810101	Steric parameter
55	CHAM830107	A parameter of charge transfer capability
56	CHAM830108	A parameter of charge transfer donor capability
57	EISD860101	Solvation free energy
58	FAUJ880104	Length of the side chain
59	FAUJ880109	Number of hydrogen bond donors
60	FAUJ880111	Positive charge
61	FAUJ880112	Negative charge
62	GARJ730101	Partition coefficient
63	GUYH850101	Partition energy
64	HUTJ700101	Heat capacity
65	HUTJ700102	Absolute entropy
66	LEVM760105	The radius of gyration of the side chain
67	MEIH800103	Average side chain orientation angle
68	VELV850101	Electron-ion interaction potential
69	TAKK010101	Side-chain contribution to protein stability (In kJ/mol)
70	ZHOH040102	Relative stability scale

Supplementary Table S2. List of features collected from web servers and sequences.

Serial No.	Features	Webserver/ Method
1	Exposed residue percentage	NetSurfP
2	Relative solvent accessibility (RSA)	NetSurfP
3	Absolute solvent accessibility (ASA)	NetSurfP
4	Probability for α-helix	NetSurfP
5	Probability for β-strand	NetSurfP
6	Probability for coil	NetSurfP
7	Number of Hot Spots (nHS)	AGGRESCAN
8	Area of the profile above threshold (AAT)	AGGRESCAN
9	Total hot spot area (THSA)	AGGRESCAN
10	Total area (TA)	AGGRESCAN
11	Normalized aggregation propensity (Na4vSS)	AGGRESCAN
12	Aromatic composition	Sequence Composition
13	Polar composition	Sequence Composition
14	Non-polar composition	Sequence Composition
15	Charge composition	Sequence Composition
16	Positive charge composition	Sequence Composition
17	Negative charge composition	Sequence Composition
18	Symmetric charge[#]	PAGE[*]
19	Aromaticity	PAGE[*]
20	β-sheet propensity	PAGE[*]

* Features are taken from PAGE, a mathematical model that predicts the aggregation rate of amyloidogenic proteins [Tartaglia et al., 2005]

Residues considered to calculate the composition (single-letter amino acid code):

Aromatic:	Y, F, W
Non-polar:	A, G, I, L, M, P, V
Polar:	R, N, D, C, Q, E, H, K, S, T
Charged:	R, K, D, E
Positively charged:	R, K
Negatively charged:	D, E

[#] **Symmetric charge residues**: opposite charges that are symmetrically placed with respect to the central amino acid in the sequence.

Supplementary Table S3. Sequence conservation calculated for the AL-Base dataset using Shannon entropy (H).

Region	Sequence conservation*	All data		Kappa (κ)		Lambda (λ)	
		AL	Non-AL	AL	Non-AL	AL	Non-AL
Variable (V_L)	Low	29.4	19.3	8.4	12	16.5	16.5
	Medium	32.1	45	20.6	28.7	33.9	30.3
	High	38.5	35.8	71	59.2	49.5	53.2
FR	Low	15.9	7	2.2	3.4	5.7	6.8
	Medium	36.4	47.7	20.2	29.2	33	28.4
	High	47.7	45.3	77.5	67.4	61.4	64.8
CDR	Low	85.7	65.2	38.9	52.6	61.9	57.1
	Medium	14.3	34.8	22.2	26.3	38.1	38.1
	High	0	0	38.9	21	0	4.8

* The interpretations are based on the Shannon entropy (H) value:

H>=2.0: Low

H<2.0 and H>1.0: Medium

H<=1: High

Note: Shannon entropy is calculated for each sequence position in multiple sequence alignment, which has more than 50% occupancy to remove the rare insertion/deletion.

AL represents amyloidogenic light chain dataset and Non-AL represents non-amyloidogenic light chain dataset.

Supplementary Table S4. Statistical analysis of the aggregation related features.

Segment of antibody	Hydrophobicity			% Gatekeeper residues			Disorderness		
	**AL#**	**Non-AL#**	**p-value***	**AL#**	**Non-AL#**	**p-value***	**AL#**	**Non-AL#**	**p-value***
**V_L-region**	0.13 ± 0.04	0.11 ± 0.04	1.49×10^{-15}	19.7 ± 2.10	21.13 ± 2.38	5.73×10^{-26}	**0.36 ± 0.07**	**0.32 ± 0.06**	**5.42×10^{-18}**
**FR1**	0.12 ± 0.08	0.12 ± 0.07	0.29	**15.66 ± 4.46**	**19.24 ± 4.47**	**7.43×10^{-36}**	0.46 ± 0.09	0.41 ± 0.09	4.34 × 10-17
**FR2**	0.14 ± 0.11	0.12 ± 0.12	0.0003	**21.65 ± 5.38**	**24.23 ± 5.32**	**6.63×10^{-15}**	0.34 ± 0.1	0.3 ± 0.08	4.54 × 10-14
**FR3**	0.1 ± 0.09	0.14 ± 0.08	2.79×10^{-16}	**24.18 ± 5.33**	**24.12 ± 5.13**	**0.85**	0.39 ± 0.09	0.34 ± 0.08	1.37 × 10-22
**FR4**	0.29 ± 0.23	0.13 ± 0.25	1.27×10^{-25}	**15.75 ± 11.83**	**24.67 ± 12.52**	**1.05×10^{-31}**	0.25 ± 0.08	0.28 ± 0.10	1.60 × 10-10
**CDR1**	**0.10 ± 0.2**	**0.007 ± 0.2**	**1.16×10^{-14}**	12.12 ± 11.67	9.04 ± 11.75	1.23×10^{-05}	0.25 ± 0.08	0.22 ± 0.08	3.92 × 10-09
**CDR2**	**0.09 ± 0.42**	**0.12 ± 0.43**	**0.16**	33.84 ± 26.52	18.95 ± 21.2	1.53×10^{-20}	0.45 ± 0.14	0.38 ± 0.11	3.78 × 10-17
**CDR3**	**0.16 ± 0.26**	**0.05 ± 0.29**	**5.23×10^{-13}**	14.94 ± 9.7	15.56 ± 8.98	0.28	0.22 ± 0.08	0.19 ± 0.08	4.41 × 10-06

* p-values obtained from t-test.

# The mean values are presented with the standard deviation for amyloidogenic (AL) and non-amyloidogenic light chain (non-AL) dataset.

Note: The biologically relevant segments for the respective features are highlighted in the table.

Supplementary Figure S2. Hydrophobicity of CDRs plotted against the percentage of gatekeeper residues in FRs for amyloidogenic (♦) and non-amyloidogenic light chain (□) dataset.

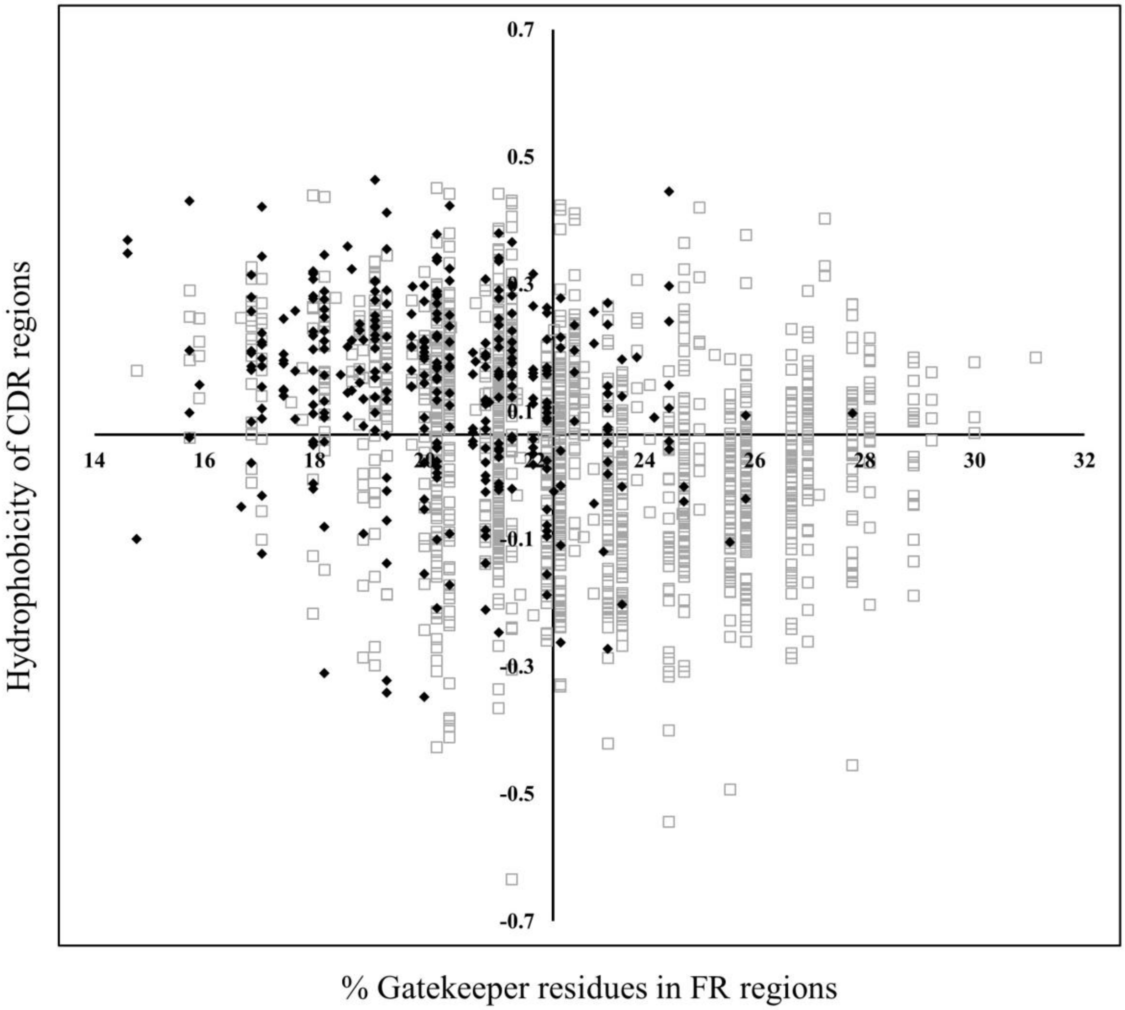

The amyloidogenic light chains are mainly present in the second quadrant (high hydrophobicity and low percentage of gatekeeper residues). The second quadrant contains 63.8% of the amyloidogenic light chains and 29% of the non-amyloidogenic light chains.

The average values of "hydrophobicity of CDR region" and "percentage of gatekeeper residues for the FR region" were considered the origin of the plot.

Supplementary Figure S3. Correlation between the features selected for the final model.

	Hydrophobicity of the CDRs	Gatekeeper percent in FRs	Disorderness	β-propensity of the V-region	Non-polar residue composition	Charge transfer capability of CDRs	Transfer free energy to surface for FRs
**Hydrophobicity of the CDRs**	1						
**Gatekeeper percent in FRs**	-0.3	1					
**Disorderness**	0.12	-0.22	1				
**β-propensity of the V-region**	-0.09	0.14	-0.10	1			
**Non-polar residue composition**	0.37	-0.01	0.19	0.03	1		
**Charge transfer capability of CDRs**	0.43	-0.4	0.64	-0.13	0.43	1	
**Transfer free energy to surface for FRs**	0.33	-0.59	0.67	-0.13	0.08	0.56	1

Supplementary Figure S4. Analysis of four additional features selected in the classification model for amyloidogenic (red) and non-amyloidogenic light chain (blue) dataset.

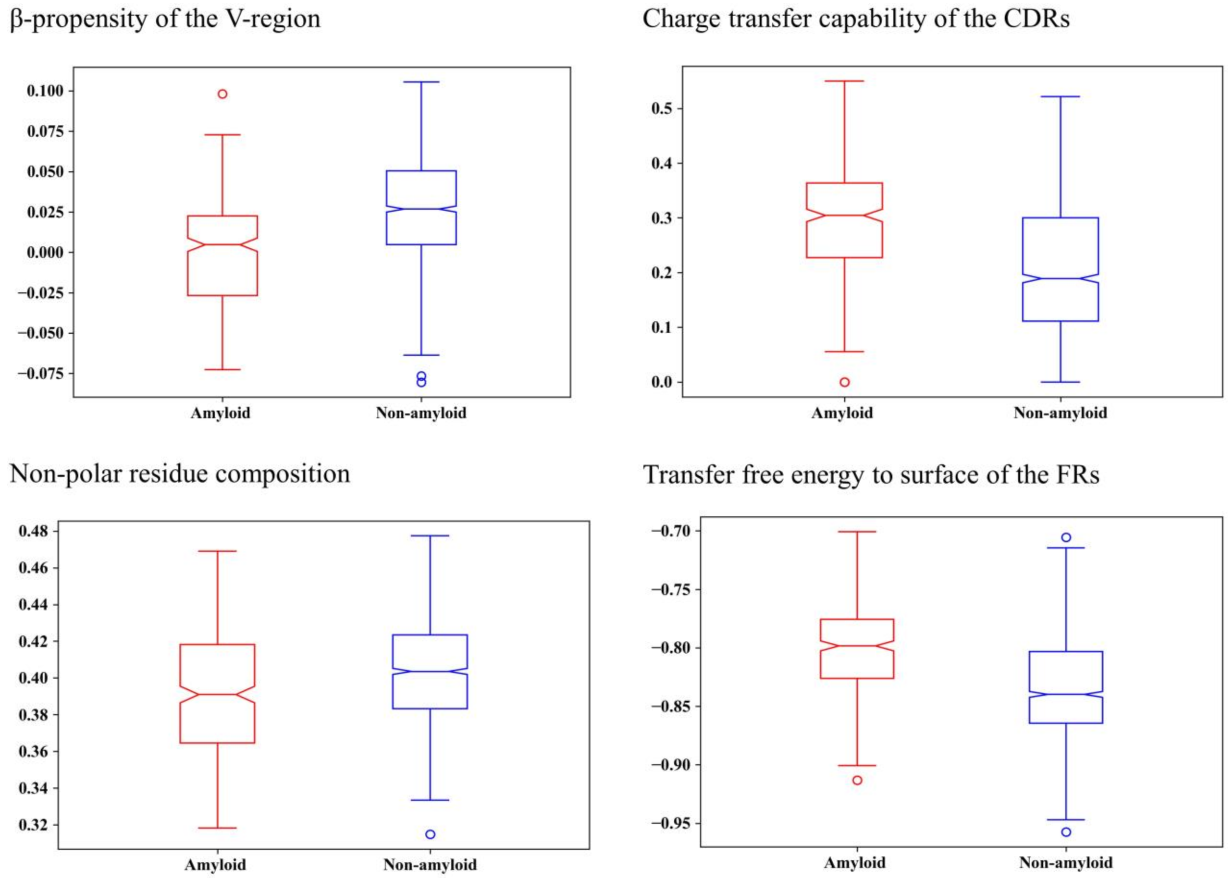

Note: β-propensity feature calculates the fraction of residues favoring the β-strand conformation (calculated as given in Tartaglia et al., 2005)

The p-values for β-propensity of the V-region (6.76×10^{-41}), non-polar residue composition (8.81×10^{-06}), Charge transfer capability of the CDRs (1.1×10^{-22}) and Transfer free energy to surface of the FRs (8.81×10^{-37}) were obtained from two-tailed t-test.

Supplementary Table S5. The importance of the features measured after removing one feature or using one feature at a time.

	**Accuracy**	**Sensitivity**	**Specificity**	**Accuracy2$**	**ROC**
Training dataset (self-consistency)	81.9	82.4	81.8	82.1	0.9
**Upon removal of one feature at a time***					
Hydrophobicity of the CDRs	84.1	78	85.5	81.8	0.89
Gatekeeper percent in FRs	67.1	92.3	61.1	76.7	0.86
Disorderness	75.2	89.1	71.9	80.5	0.89
β-propensity of the V-region	76.9	85.9	74.8	80.4	0.89
Non-polar residue composition	79.2	83.1	78.4	80.8	0.89
Charge transfer capability of CDRs	73.3	89.8	69.4	79.6	0.86
Transfer free energy to surface for FRs	75.2	91.1	71.5	81.3	0.89
**Using single feature at a time#**					
Hydrophobicity of the CDRs	62.8	64.9	62.4	63.6	0.64
Gatekeeper percent in FRs	55.4	93.3	46.5	69.9	0.77
Disorderness	71.5	46.6	77.3	62	0.63
β-propensity of the V-region	54	87.5	46.1	66.8	0.73
Non-polar residue composition	67.2	52.7	70.6	61.6	0.63
Charge transfer capability of CDRs	72.1	54	76.4	65.2	0.68
Transfer free energy to surface for FRs	57.6	85.3	51.1	68.2	0.72

Note: The importance of features is calculated at selected model threshold (0.15) on training dataset and measured using area under the ROC curve.

* The performance of the model measured upon removing one feature at a time to evaluate the effect on self-consistency.

# The performance of the model measured using only one feature at a time to evaluate the predictive capability of the respective feature.

$ $Accuracy2 = \frac{Sensitivity+Specificity}{2}$

Performance of aggregation-prone region prediction algorithm

Aggregation propensity and aggregation-prone regions

We have analyzed the performance of the aggregation-prone region prediction algorithm, such as TANGO [Fernandez-Escamilla et al., 2004] and WALTZ [Maurer-Stroh et al., 2010] on AL-Base dataset. Surprisingly, aggregation propensity predicted by both algorithms showed slightly lower values for the amyloidogenic light chain dataset compared to the non-amyloidogenic light chain dataset (**Supplementary Figure S5 (a)**). A further classification of the antibody dataset to kappa (κ) and lambda (λ) isotype showed similar results for TANGO algorithm (**Supplementary Figure S5 (b)**). However, WALTZ prediction for the lambda (λ) dataset showed a higher aggregation propensity for the amyloidogenic light chain dataset (**Supplementary Figure S5 (c)**). Since there was no significant variation in the aggregation propensity of light chain variable region (V_L) in amyloidogenic and non-amyloidogenic datasets, we further analyzed the aggregation propensity of aggregation-prone regions (APRs) (**Supplementary Figure S6**). Similar to variable region analysis, aggregation propensity of the APRs also presented ambiguous results. TANGO predicted a slightly higher aggregation propensity for the amyloidogenic light chains, where WALTZ had almost similar aggregation propensity for amyloidogenic and non-amyloidogenic light chains (**Supplementary Figure S6 (a)**). For the kappa (κ) isotype, TANGO predicted a higher aggregation propensity for the amyloidogenic light chains; however, WALTZ predicted lower aggregation propensity. Lambda (λ) isotype had almost similar aggregation propensity predicted by TANGO and WALTZ for amyloidogenic and non-amyloidogenic light chains (**Supplementary Figure S6 (b,c)**).

We further analyzed the position and average aggregation propensity of the APRs present in the variable region of the light chain (V_L region) (**Supplementary Table S6, Supplementary Figure S7**). WALTZ (95.4% AL-Base light chain sequences) predicted almost 3.4 times more APRs in the complete AL-Base dataset compared to TANGO (45.7% AL-Base light chain sequences) (**Supplementary Table S6)**. Most of the APRs predicted by both the algorithms were present in the CDR1-FR2, FR2-CDR2 and FR3 in the V_L region (**Supplementary Figure S7**). TANGO also predicted significant APRs in the CDR3-FR4. WALTZ predicted more APRs and higher average APR aggregation propensity in the amyloidogenic light chains of CDR1-FR2 (percentage of APR: 48% for amyloid and 43.1% for non-amyloid; average aggregation propensity of APRs: 71.5 for amyloid and 68.5 for non-amyloid) and FR2-CDR2 (percentage of APR: 37.8% for amyloid and 28.4% for non-amyloid; average aggregation propensity of APRs: 55.7 for amyloid and 50.2 for non-amyloid). However, TANGO showed a higher average aggregation propensity for the non-amyloidogenic light chains in CDR1-FR2 (42.4 for amyloid and 44.7 for non-amyloid) and FR2-CDR2 (53.2 for amyloid and 56.6 for non-amyloid). The percentage of APRs were higher in the amyloidogenic light chains for CDR1-FR2 (35% for amyloid and 32.3% for non-amyloid) and lower in FR2-CDR2 (21.4% for amyloid and 32% for non-amyloid). TANGO has also predicted APRs in CDR3-FR4 region where amyloidogenic light chains have more APRs (23.6% for amyloid and 11.6% for non-amyloid) with higher average aggregation propensity (56.7 for amyloid and 46.7 for non-amyloid).

Analysis of gatekeeper residues

There is a high variation in the number of APRs, APR position and aggregation propensity predicted by TANGO and WALTZ. However, both algorithms predicted APRs in amyloidogenic as well as in non-amyloidogenic light chain dataset. The presence of the gatekeeper residues in the flanks of the APRs can substantially discourage the amyloid formation. Hence, we further analyzed the presence of gatekeeper residues in ±3 residue flanks of the APRs (**Supplementary Table S7**). The gatekeeper analysis shows that most of the APRs in light chain dataset contains gatekeeper in one flank and non-amyloidogenic light chain dataset have a significantly higher percentage of such APRs compare to amyloidogenic light chain dataset. There are also a higher number of APRs with no gatekeeper in ±3 residue flanks in the amyloidogenic light chain dataset.

Analysis of FR3 region

Interestingly, TANGO and WALTZ predicted a significant amount of APRs in the relatively buried and conserved FR3 region. Our analysis in **section 3.2.2** in the main text (**Figure 2 (b)**) has already shown that the FR3 region has a relatively higher percentage of gatekeeper residues compared to other regions. It is also the only region with almost equal percentage of gatekeeper residues in amyloidogenic and non-amyloidogenic light chain datasets. It suggests that APR regions are inherently present in FR3 regions and subdued by a higher percentage of gatekeeper residues. We further checked the presence of gatekeeper residues in the ±3 residue flank of the predicted APRs in amyloidogenic and non-amyloidogenic light chain dataset. The amyloidogenic light chains had a significantly higher number of APRs predicted by TANGO (amyloid: 45% and non-amyloid: 23.2%) and WALTZ (amyloid: 47.1% and non-amyloid:6.8%) which does not contain any gatekeeper residues in the ±3 residue flanks. The analysis shows that FR3 regions, if exposed, might be potential nucleating regions in the light chain variable region of antibodies and the position of the gatekeeper residues relative to APR(s) in the FR3 region can play an important role in the aggregation process.

Supplementary Figure S5. Aggregation propensity for the amyloidogenic (red) and non-amyloidogenic light chain (blue) dataset predicted by TANGO and WALTZ. (a) all variable region sequences predicted using TANGO and WALTZ, (b) variable region isotypes (kappa and lambda) predicted by TANGO, (c) variable region isotypes (kappa and lambda) predicted by WALTZ.

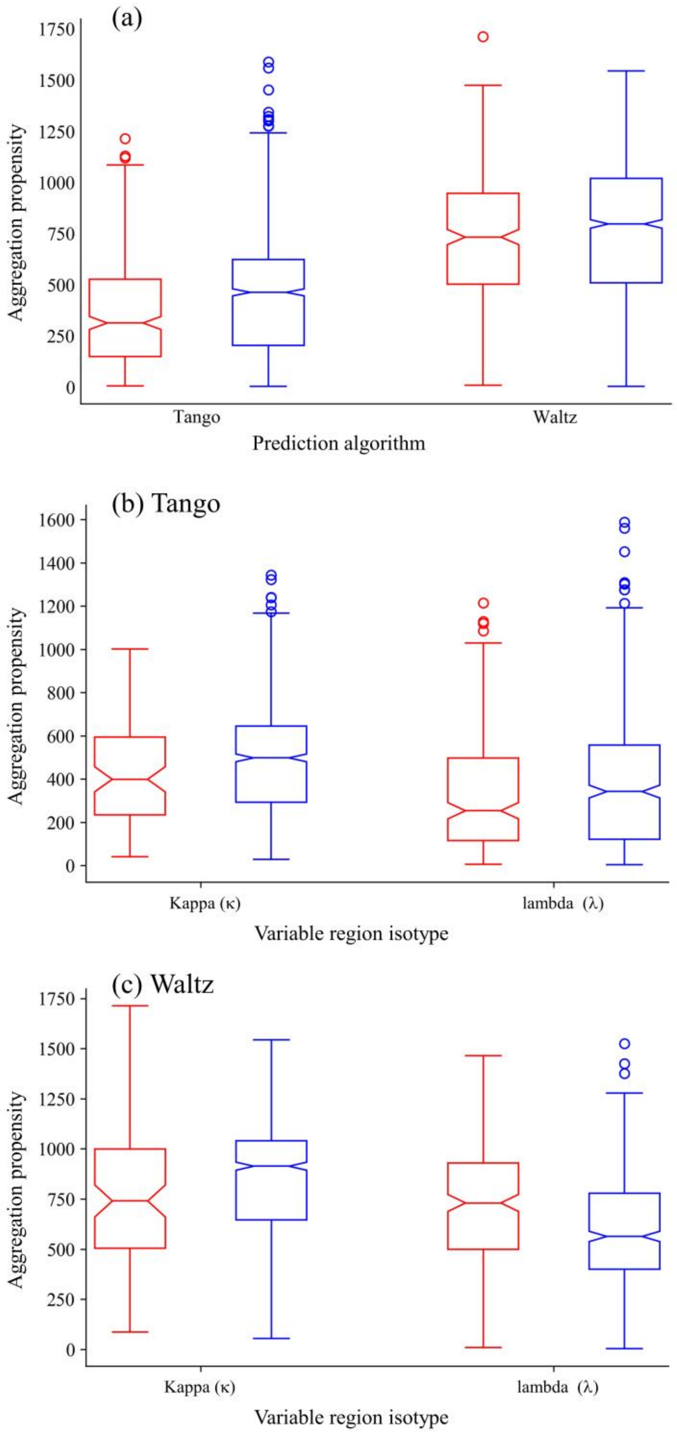

Supplementary Figure S6. Aggregation propensities of the aggregation-prone regions (APRS) predicted for the amyloidogenic (red) and non-amyloidogenic light chain (blue) dataset using TANGO and WALTZ. The aggregation propensity of the APRs predicted for (a) complete dataset using TANGO and WALTZ, (b) variable region isotypes (kappa and lambda) using TANGO, (c) variable region isotypes (kappa and lambda) using WALTZ.

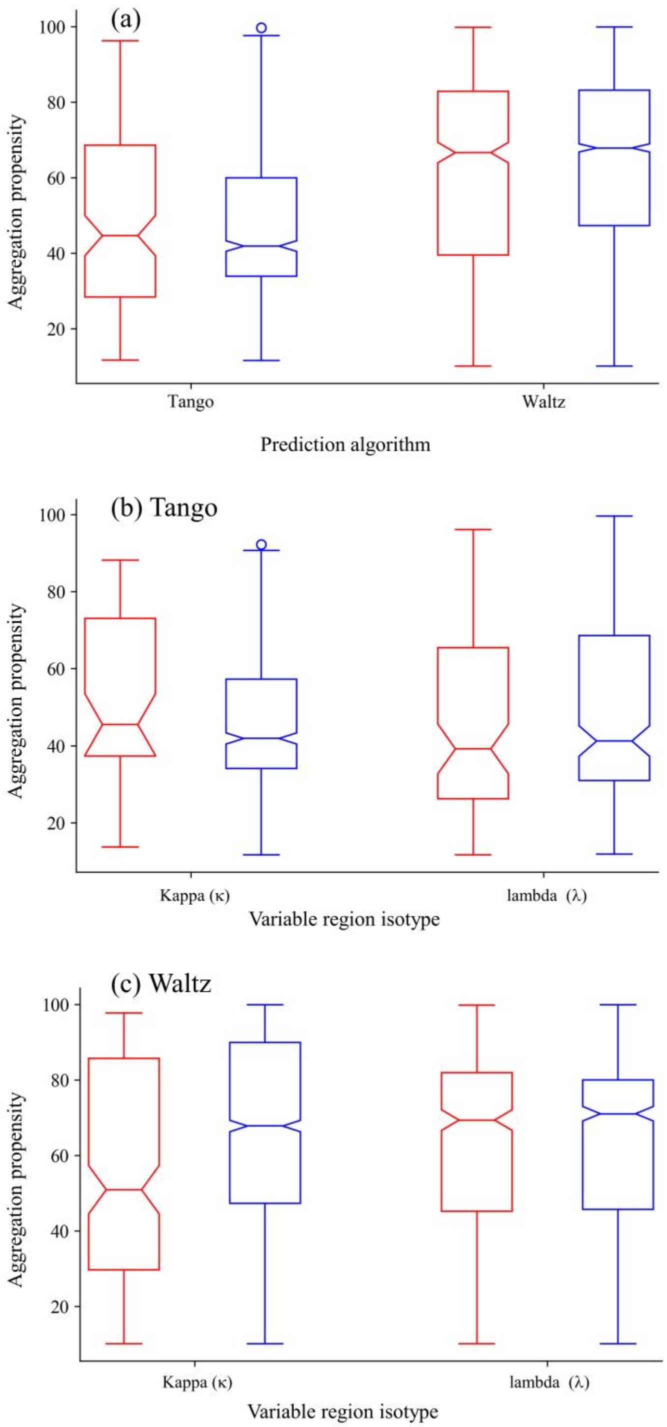

Supplementary Figure S7. Percentage of aggregation-prone regions (APRs) predicted by TANGO and WALTZ in each segment of light chain variable region of antibodies and their respective average aggregation propensity.

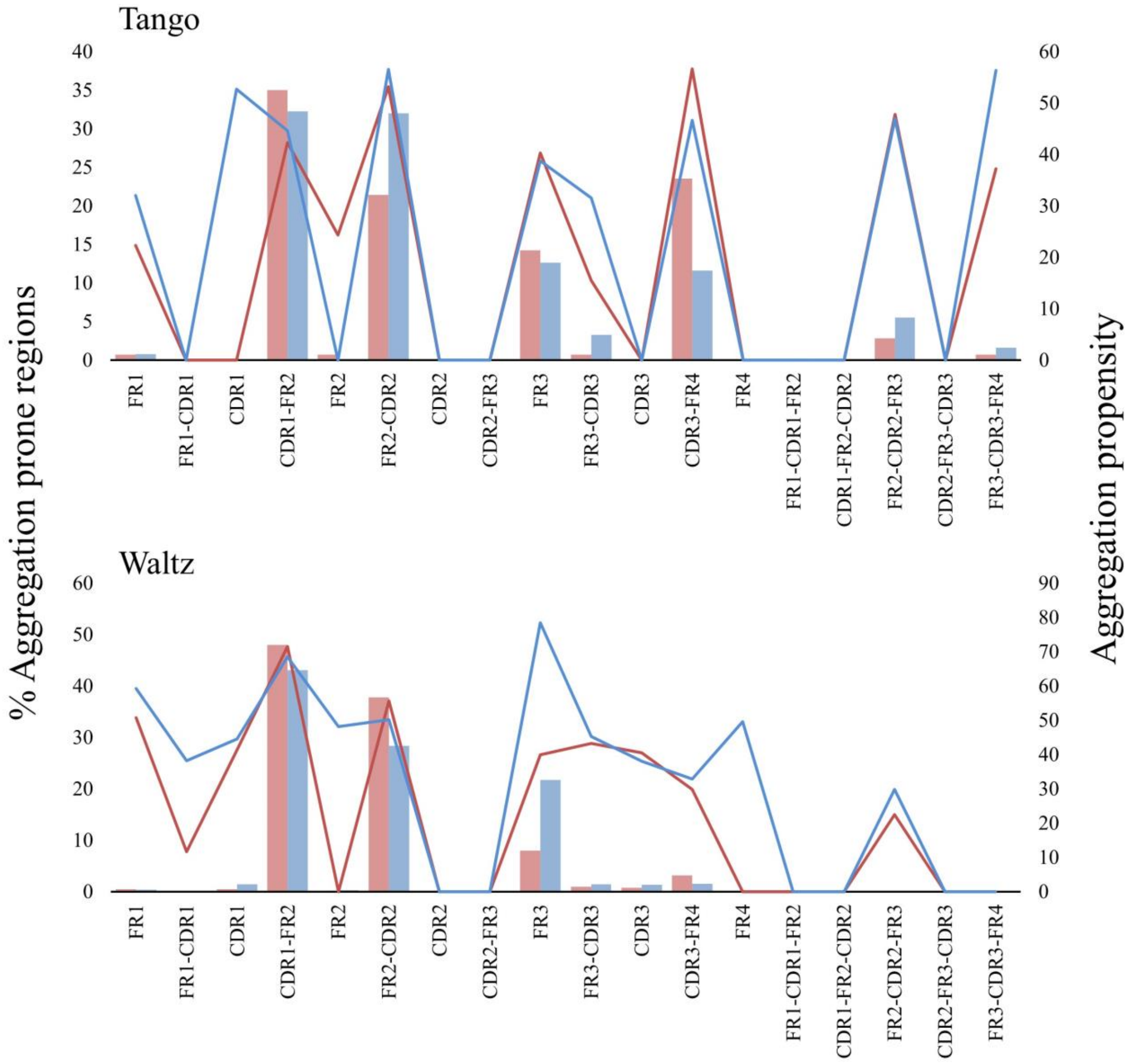

Note: Bar graph shows the percentage of the APRs present in the respective segment (left side y-axis) and the line plot shows that average aggregation propensity of the APRs in that segment (right side Y-axis) of the light chain variable region of the antibody, for amyloidogenic (red) and non-amyloidogenic light chain (blue) dataset.

Supplementary Table S6. Comparison of aggregation-prone region prediction algorithms (TANGO and WALTZ).

Method	Sequence length		Aggregation propensity (sequence)		Total predicted APRs	Unique APRs	Aggregating sequences*
	Average	Range	Average	Range			
**TANGO**							
**AL**	109.5	106-114	354.6	5.4-1215	140	113	120 (34.5%)
**Non-AL**	109.3	102-120	446.7	3.2-1588.4	852	335	715 (48.3%)
**Overlap#**						24	
**WALTZ**							
**AL**	109.5	106-114	717.8	9.3-1713.4	635	254	334 (96%)
**Non-AL**	109.3	102-120	753.9	2.8-1544.2	2730	441	1409 (95.2%)
**Overlap#**						104	

* Sequences with at least one APR present.

# overlapping non-redundant APRs in AL and Non-AL dataset, predicted by the respective algorithm.

Note: AL represents amyloidogenic light chain dataset and Non-AL represents non-amyloidogenic light chain dataset.

Supplementary Table S7. Analysis of the gatekeeper residues present in ±3 flanks of the APRs.

	**APRs**			**Aggregating Sequences***
	**Gatekeepers on both flank**	**Gatekeeper at one flank**	**APRs with no gatekeeper**	
**TANGO**				
AL	38 (27.1%)	63 (45%)	39 (27.8%)	29 (24.2%)
Non-AL	246 (28.9%)	528 (62%)	78 (9.2%)	61 (8.5%)
**WALTZ**				
AL	248 (39%)	339 (53.4%)	48 (7.6%)	1 (0.3%)
Non-AL	713 (26.1%)	1931 (70.7%)	86 (3.1%)	6 (0.4%)

***** Light chain **v**ariable region sequences that contain at least one predicted APR from the respective algorithm but do not contain gatekeeper residues in the ±3 residue flank.

Note: AL is amyloidogenic light chain dataset and Non-AL is non-amyloidogenic light chain dataset

References

1. Gromiha, M. M., Oobatake, M., & Sarai, A. (1999). Important amino acid properties for enhanced thermostability from mesophilic to thermophilic proteins. Biophys Chem, 82(1), 51-67.
2. Kawashima, S., & Kanehisa, M. (2000). AAindex: amino acid index database. Nucleic acids research, 28(1), 374-374.
3. Tartaglia, G. G., Cavalli, A., Pellarin, R., & Caflisch, A. (2005). Prediction of aggregation rate and aggregation-prone segments in polypeptide sequences. Protein Science, 14(10), 2723-2734.
4. Fernandez -Escamilla, A. M., Rousseau, F., Schymkowitz, J., & Serrano, L. (2004). Prediction of sequence-dependent and mutational effects on the aggregation of peptides and proteins. Nature biotechnology, 22(10), 1302.
5. Maurer-Stroh, S., Debulpaep, M., Kuemmerer, N., De La Paz, M. L., Martins, I. C., Reumers, J., Morris, K. L., & Schymkowitz, J. W. (2010). Exploring the sequence determinants of amyloid structure using position-specific scoring matrices. Nat Methods, 7(3), 237.
